# Stability of Hybrid Organic-Inorganic Perovskite CH_3_NH_3_PbBr_3_ Nanocrystals under Co-Stresses of UV Light Illumination and Temperature

**DOI:** 10.3390/nano9081158

**Published:** 2019-08-13

**Authors:** Weijie Guo, Nan Chen, Binbin Xu, Yijun Lu, Bin Li, Tingzhu Wu, Qijin Cheng, Yang Li, Jin Chen, Yue Lin, Zhong Chen

**Affiliations:** 1 Fujian Engineering Research Center for Solid-State Lighting, Collaborative Innovation Center for Optoelectronic Semiconductors and Efficient Devices, Department of Electronic Science, Xiamen University, Xiamen 361005, China; 2 College of Chemistry and Chemical Engineering, Xiamen University, Xiamen 361005, China; 3 School of Science, Nanjing University of Posts and Telecommunications, Nanjing 210023, China; 4 College of Energy, Xiamen University, Xiamen 361102, China; 5 State Key Laboratory for the Physical Chemistry of Solid Surfaces, Xiamen University, Xiamen 361005, China

**Keywords:** perovskite nanocrystals, stability, combined stresses, electronic structure, photophysical properties

## Abstract

Hybrid organic–inorganic metal halide perovskite nanocrystals (NCs) are among the candidates for color conversion materials in displays, especially in NC-based micro-light-emitting diode (micro-LED) displays. However, these NCs are still lacking long-term stability, which has hindered their large-scale applications. We mimic the working conditions, which include ultraviolet light illumination at 323 K and three different types of atmosphere (N_2_, vacuum, and air), respectively, to investigate the stability of CH_3_NH_3_PbBr_3_ NCs embedded in the polyvinylidene fluoride matrix. X-ray diffraction results indicate the generation of NH_4_Pb_2_Br_5_, which is produced from the encapsulated CH_3_NH_3_PbBr_3_ NCs in all three atmospheres, and the decomposition generates a large amount of accompanying interface defects at the surface area of NCs, resulting in the significant decrease of the photoluminescence (PL) intensity. This work highlights the stability-related mechanism of CH_3_NH_3_PbBr_3_ NCs under combined external stresses that mimic operating conditions. In addition, this work also suggests a new method for conducting aging tests and contributes to developing effective routes towards higher stability of perovskite NCs.

## 1. Introduction

Hybrid organic-inorganic perovskites (HOIPs) have attracted great interest because they exhibit unique optical properties when functioning as luminescent materials for optoelectronic devices [1,2,3,4]. Under high-energy light excitations, HOIP nanocrystals (NCs) provide bright and narrow-band photoluminescence (PL), the peak wavelength of which is easily tunable within the visible spectral range by simply adjusting the mixing ratio of different halide ions. In addition, HOIP NCs can be synthesized in facile and low-cost manners, such as the solution-processing method to obtain perovskite nanocrystals embedded in a polymer matrix [5]. These two advantages make HOIP NCs highly promising in display and solid-state lighting applications by offering a wide color gamut or high color-rendering index [1,6,7]. In these applications, HOIP NCs serve as photon energy down conversion materials, transforming the blue light emitted from the InGaN light-emitting diodes (LEDs), or ultraviolet (UV) light from the AlGaN LEDs, into lower-energy emissions. These narrow and tunable spectra constitute highly saturated blue, green, and red primary colors, facilitating the fabrication of full spectrum white LEDs or full-color micro-light-emitting diode (micro-LED) displays [1,2,7,8,9]. Incorporating the down conversion materials emitting red, green, and blue colors onto the UV micro-LED array has been demonstrated as a feasible solution for the micro-LED display [7,8,10]. While the optoelectronic properties of HOIP NCs have been widely investigated, the issue of their long-term stability under the combined effects of light, moisture, and oxygen poses a great challenge to their commercialization [1,3,11]. Investigating the fundamental instability mechanisms of HOIP NCs and uncovering the origin of the instability are therefore of paramount importance [4].

Extensive studies on methylammonium lead halide perovskites, CH_3_NH_3_PbX_3_ (MAPbX_3_, X=Cl, Br, or I), through experiments or theoretical calculations, have shed light on the mechanisms of instability [3,12,13,14]. According to the Raman spectra of MAPbI_3_ with different domain sizes, four stages exist before MAPbI_3_ fully decomposes into PbI_2_ [11]. The transition between stages I and II under short-term light illumination is reversible; however, the exact mechanism has not been determined [11]. The stability issue of MAPbX_3_ remains an open question before the decomposition mechanism can be further clarified [13]. Encapsulating MAPbX_3_ via proper surface passivation or functionalization can block molecular desorption, hinder the infiltration of external species into the perovskite lattice, and reduce chemical interactions with the surrounding environment, therefore enhancing its stability [2,9,13,15]. However, the enhancement in stability through the protection of barrier materials or chemical passivation is limited, as light irradiation alone can expedite the decomposition process. Down-converting materials, employed in displays or solid-state lighting, should withstand the illumination from LED chips, usually blue or UV light, and the heat that originates from nonradiative processes during wavelength conversion. While the thermodynamic analysis suggests that thermal decomposition of MA^+^ is largely favored for all MAPbX_3_ compounds, only the decomposition in MAPbI_3_ has been experimentally observed [16,17]. During light soaking, the ion migration in MAPbI_3_ film can induce the accumulation of iodide ions [18], which might be suppressed in NCs embedded in polymer due to the blocking of capping surfactants or matrix. Above 323 K, the worsening of the optical properties of MAPbI_3_ becomes rapid and irreversible [19]. The breakage of C-N bonds in MA^+^ has been demonstrated upon annealing of MAPbI_3_ films on an indium tin oxide substrate for 24 h in the dark at 358 K in different atmospheres (N_2_, O_2_, and air atmosphere with 50% relative humidity) [20]. For MAPbBr_3_ NCs, it has been reported that synergistic effects from moisture and illumination cause apparent instability [15], while the synergistic effects of thermal and illumination remain to be investigated. In addition, a large body of past research has focused on the dissociation at the surface of MAPbX_3_ film or crystal [21], whereas studies on green-emitting MAPbBr_3_ NCs in the matrix subjected to light illumination and heat are limited.

In this work, we investigate the original mechanism of MAPbBr_3_ NCs embedded in polyvinylidene fluoride (PVDF) film (MBNCs-PVDF) in different atmospheres under the illumination of a UV LED and at a temperature of 323 K, which is the suggested testing temperature for NCs to be used in displays [1]. The transformation from perovskite to tetragonal NH_4_Pb_2_Br_5_ is evidenced by the X-ray diffraction (XRD) results, suggesting a structural instability in MAPbBr_3_ NCs. Furthermore, this work was conducted under an illumination and temperature condition that is relevant to the aging in real devices, thus offering a better insight for improving the stability or evaluating the properties of MAPbX_3_ for use in optoelectronic applications.

## 2. Materials and Methods 

**Materials.** Polyvinylidene fluoride (PVDF, ZZBIO Co., Ltd., Shanghai, China), PbBr_2_ (99.99%, Xi’an Polymer Light Technology Corp., Xi’an, China), CH_3_NH_3_Br (methylammonium bromide, 99.5%, Xi’an Polymer Light Technology Corp.), and *N*,*N*-dimethylformamide (analytical grade, Sinopharm Chemical Reagent Co., Ltd., Shanghai, China).

**Figure 1:**
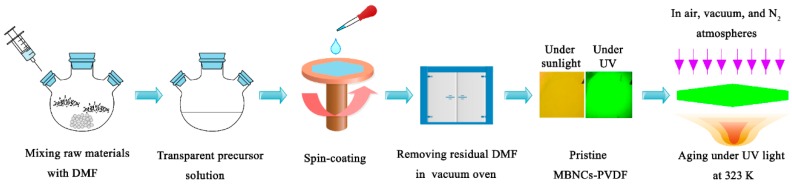
The scheme of the experiments.

**Preparation of MBNCs-PVDF.** MBNCs-PVDF are synthesized in an argon-filled glovebox, following the in situ fabrication method illustrated in Figure 1 [2]. The mixture of CH_3_NH_3_Br (0.08 mmol), PbBr_2_ (0.1 mmol), PVDF (0.84 g), and DMF (5 mL) is stirred at 600 r/min for 24 h at room temperature to achieve a transparent precursor solution. Then, a thin layer of the precursor solution is obtained by spin-coating (2000 r/min, 25 s) on the surface of a flat glass plate. After heating the glass substrate with precursor solution on a hot plate at 303 K, we obtain a solid film attached on the surface of glass. Put the glass substrate with solid film into a vacuum oven at 303 K for 24 h to further remove the residual DMF. Finally, the solid film, MBNCs-PVDF, is peeled off from the glass substrate for characterization.

**Aging under Co-stresses.** The MBNCs-PVDF film is fixed at one end of a hollow cylinder, while at the other end a 368 nm UV LED, powered by small battery containing dry cells, functioning as the excitation source. The UV light emitted from the LED goes through the inner of the cylinder, yielding an irradiation intensity of 5.6 mW/cm^2^ at the surface of MBNCs-PVDF. The operations of accelerated aging are conducted in an oven (SLZK-40, Shanghai Shengli Test Instruments, Co., Ltd., Shanghai, China) at 323 K under air, vacuum, and N_2_ atmosphere, respectively. Every 2 h, the MBNCs-PVDF is taken out from the oven for PL measurement at room temperature using an integrating sphere and a spectrometer (Spectro 320, Instrument Systems, Munich, Germany).

**Measurement and Characterization.** XRD are recorded by an X-ray powder diffractometer (Ultima IV, Rigaku Corporation, Tokyo, Japan) with monochromatized Cu Kα radiation. The morphology of NCs is collected by a JEOL JEM2100 transmission electron microscope (TEM, Tokyo, Japan) operating at 200 kV. The samples for TEM measurement are obtained by sectional cutting using ultra-microtome (EM UC7, Leica, Wetzlar, Germany) after mounting the MBNCs-PVDF films into epoxy resin (Epon-812). Placing the samples into an optical Cryostat (CS202E-DMX-1AL, Advanced Research Systems, Macungie, PA, USA), we collect the temperature-dependent PL spectra under the excitation of a 368 nm UV LED using a spectrometer (QE65 Pro, Ocean Optics, Largo, FL, USA).

**Calculations on Structure**. We compute electronic structures of NH_4_Pb_2_Br_5_ and CH_3_NH_3_Pb_2_Br_5_ using the full-potential linearized augmented plane wave (FP-LAPW) method implemented in the WIEN2K code [22]. The generalized gradient approximation (GGA) [23] is applied to the exchange-correlation potential calculation. The muffin tin radii are chosen to be 2.5 a.u. for Pb, Br, and N atoms, 0.65 for H atoms. The plane-wave cutoff is defined by RK_max_ = 7.0, where R is the minimum LAPW sphere radius and K_max_ is the plane-wave vector cutoff. The self-consistent calculation is performed over a 10 × 10 × 10 k-point mesh.

## 3. Results

### 3.1. PL Decay under Co-Stresses

**Figure 2:**
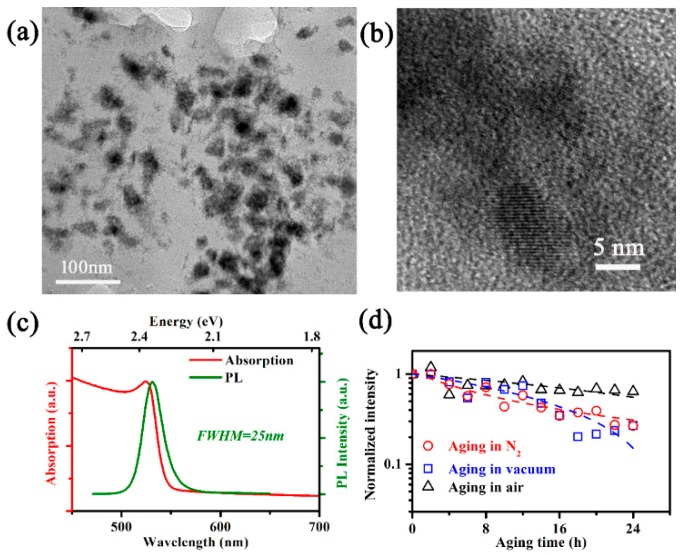
(**a**) A typical TEM image MAPbBr_3_ NC in PVDF matrix; (**b**) TEM image of a typical MAPbBr_3_ NC; (**c**) normalized absorption and PL spectrum of the fabricated MBNCs-PVDF; and (**d**) PL intensities recorded during aging, normalized to the initial intensity, and presented in logarithmic ordinate. Hollow circles correspond to aging in N_2_, hollow squares to aging in vacuum, and hollow triangles to aging in air, while dashed lines are obtained by linear fitting for aging in vacuum and air and by reciprocal fitting for aging in N_2_. The temperature and 368-nm UV light illumination density during aging are 323 K and 5.6 mW/cm^2^, respectively.

The NCs, with an average size of 15 nm (Figure 2a,b, verified by TEM), are distributed in the PVDF film, the resistances to oxygen and water of which facilitate research on the intrinsic stability of NCs [2]. At 300 K, the absorption spectrum (Figure 2c) exhibits an evident absorption peak at 2.37 eV (524 nm), while the PL peak at 2.34 eV (531 nm) has a full width at half maximum (FWHM) of 25 nm. The wavelength of PL peak of NCs in this work is shorter than those of bulk MAPbBr_3_ (545 nm) [24], whereas it is longer than that of MAPbBr_3_ quantum dots with a mean size smaller than 10 nm [25,26,27]. 

We then divide the pristine samples into three groups and subject them to 24 h aging in N_2_, vacuum and air, separately, during which the stresses are identical: 368 nm UV light illumination with an intensity of 5.6 mW/cm^2^ under a temperature of 323 K. After aging, the PL intensities of MBNCs-PVDF in vacuum and N_2_ both decrease to 25% of that of the pristine sample, and this value for the sample aged in air is 64% (Figure 2d).

### 3.2. XRD Analysis

**Figure 3:**
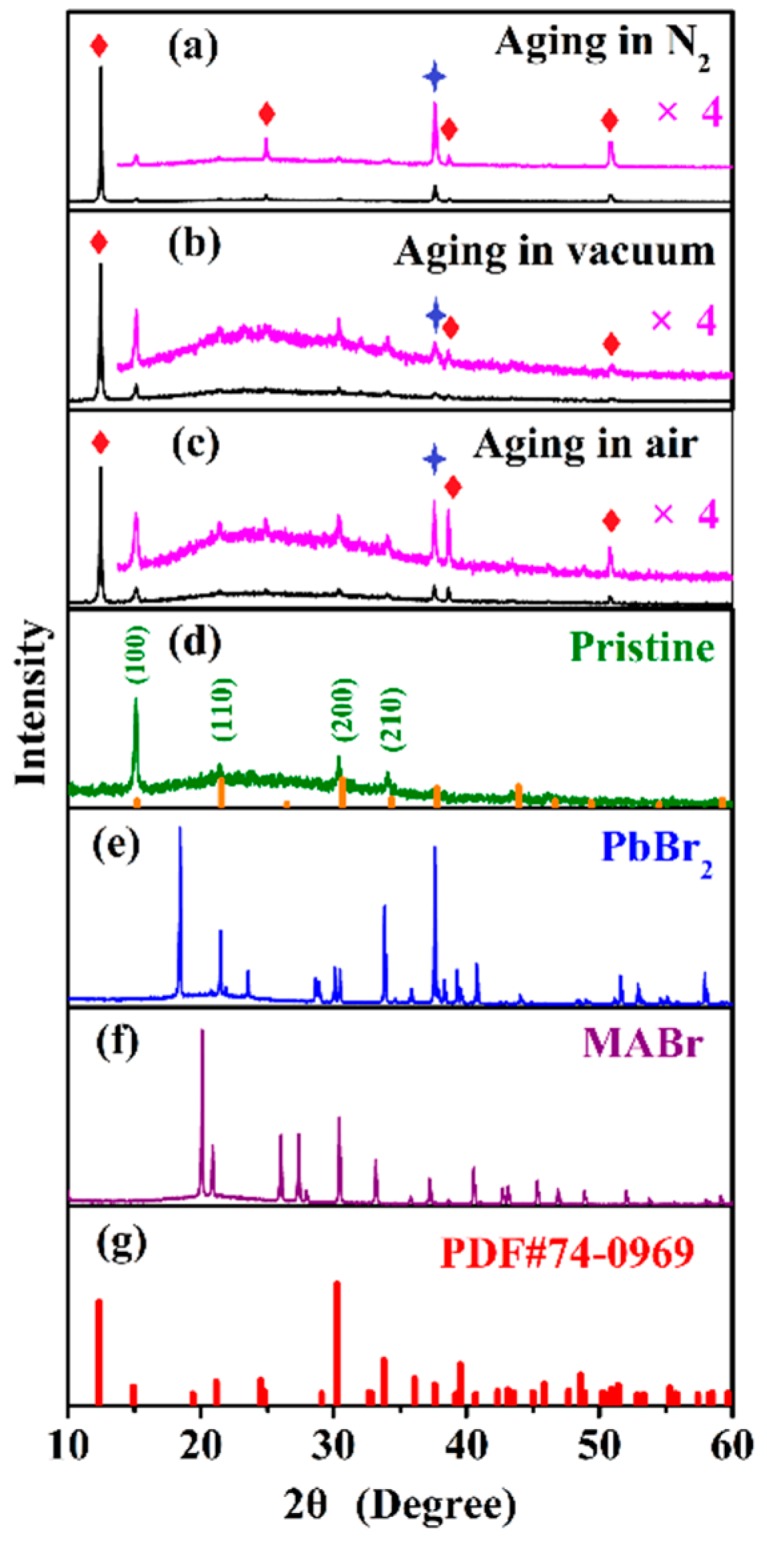
XRD patterns of MBNCs-PVDF: (**a**) After aging in N_2_; (**b**) after aging in vacuum; (**c**) after aging in an air atmosphere; and (**d**) pristine sample. For reference, (**e**) and (**f**) provide XRD patterns of PbBr_2_ and CH_3_NH_3_Br (MABr); and (**g**) provides a standard XRD pattern of NH_4_Pb_2_Br_5_ (PDF#74-0969). The standard patterns of MAPbBr_3_ (PDF#54-0752) are provided at the bottom of (**d**). Diamonds and stars indicate diffraction peaks of NH_4_Pb_2_Br_5_ and PbBr_2_, respectively. The temperature and 368-nm UV light irradiation density during aging are 323 K and 5.6 mW/cm^2^.

XRD patterns (Figure 3d) confirm the existence and structure of pristine MAPbBr_3_ NCs. The diffraction peaks at 15.3°, 21.6°, 30.5°, and 34.2° correspond to the reflections from the (100), (110), (200), and (210) crystal planes of the cubic phase structure, respectively. In addition to the diffraction peaks of pristine MBNCs-PVDF (Figure 3d), after aging in the three atmospheres, two sets of additional peaks appear in the XRD patterns (Figure 3a–c). The peak at 37.7°, corresponding to reflections from the (040) crystal plane of PbBr_2_, evidences the partial decomposition of MAPbBr_3_ NCs under the co-stresses of temperature and UV illumination, as PbX_2_ acts as a byproduct of dissociation of MAPbX_3_ under various conditions [17].

**Figure 4:**
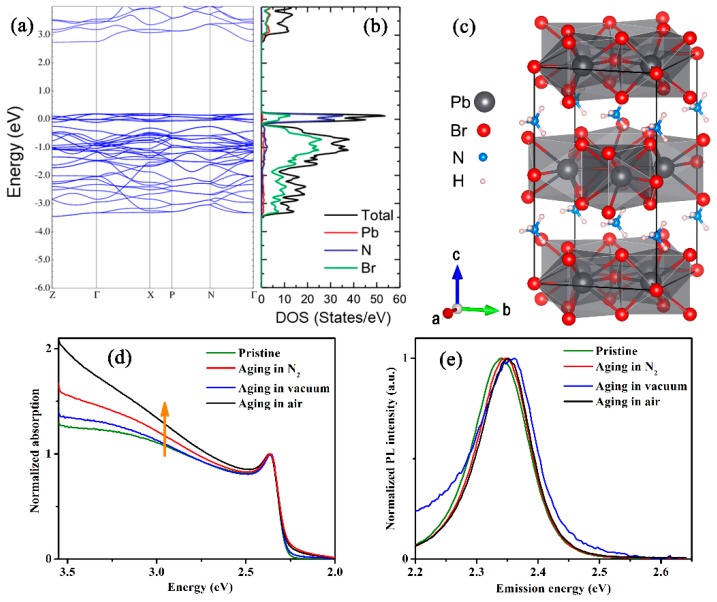
(**a**) Electronic band structure and (**b**) the density of states (DOS) of NH_4_Pb_2_Br_5_ calculated using the full-potential linearized augmented plane wave (FP-LAPW) method implemented in the WIEN2K code. (**c**) Crystal structure of tetragonal NH_4_Pb_2_Br_5_. (**d**) Normalized absorption spectra and (**e**) normalized PL spectra of the pristine MBNCs-PVDF and the samples after aging in three different atmospheres. The temperature and 368 nm UV light irradiation density during aging are 323 K and 5.6 mW/cm^2^.

Another set of diffraction peaks, including those at 12.6°, 24.8°, 38.7°, and 50.8° (Figure 3a–c), matches the reflections from the (002), (004), (321), and (008) crystal planes of NH_4_Pb_2_Br_5_ (tetragonal, I4/mcm, No. 140) [28,29]. The tetragonal phase of NH_4_Pb_2_Br_5_ exhibits a sandwich structure consisting of [Pb_2_Br_5_]^−^ layers and intercalated [NH_4_]^+^ (Figure 4c). All Pb^2+^ ions are confined in the center of the [Pb_2_Br_5_]^−^ layer, and both the bottom and top surfaces of the layer consist of Br^−^ ions. The existence of nanocrystalline NH_4_Pb_2_Br_5_ impurities has been observed in the synthesis of formamidinium lead bromide perovskite nanocrystals by synchrotron XRD [29]. 

The electronic band-structure of NH_4_Pb_2_Br_5_ has been calculated, and the result reveals that NH_4_Pb_2_Br_5_ possesses a band gap of 2.5 eV (Figure 4a,b). The purpose of the calculation on electronic structures of NH_4_Pb_2_Br_5_ is to verify the existence of NH_4_Pb_2_Br_5_ in the aged MBNCs-PVDF from optical absorption spectra, since the optical absorption should exhibit major enhancement when the photon energy beyond the bandgap of NH_4_Pb_2_Br_5_, if there exists NH_4_Pb_2_Br_5_ in the aged MBNCs-PVDF. Compared with that of the pristine MBNCs-PVDF, the aged MBNCs-PVDF exhibits conspicuous enhancement on optical absorption when the photon energy is beyond 2.5 eV (Figure 4d). Therefore, in accordance with the result of XRD, the enhanced optical absorption in the region of energy beyond 2.5 eV also indicates the existence of NH_4_Pb_2_Br_5_ of the aged MBNCs-PVDF. As we cannot observe the PL emission from NH_4_Pb_2_Br_5_ (Figure 4e), its optical properties need further investigation in view of the debate on whether the inorganic counterpart CsPb_2_Br_5_ is PL inactive or PL active [30,31].

### 3.3. Temperature-Dependent PL Spectra

**Figure 5:**
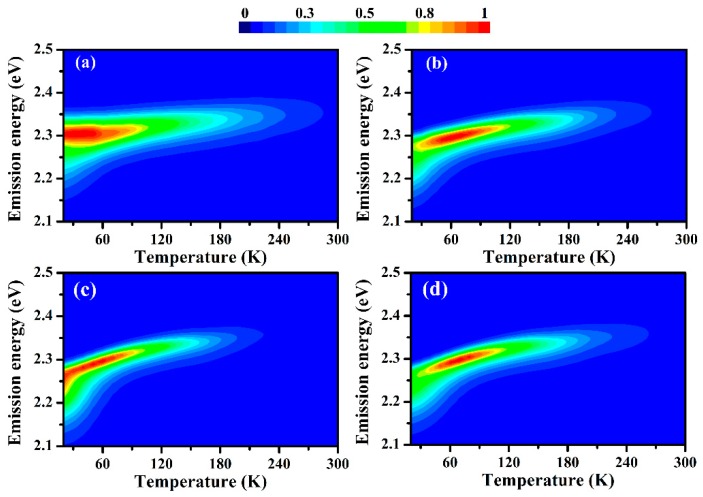
Pseudo-color maps of temperature-dependent PL spectra of MBNCs-PVDF: (**a**) Pristine; (**b**) after aging in N_2_; (**c**) after aging in vacuum; and (**d**) after aging in an air atmosphere. The aging temperature and 368 nm UV light illumination density of (**b**)–(**d**) are 323 K and 5.6 mW/cm^2^.

The temperature-dependent PL spectra are characterized to further investigate the properties of the MBNCs-PVDF before and after aging. Figure 5 depicts the pseudo-color maps of the temperature-dependent PL (TDPL) spectra of MBNCs-PVDF, pristine and after aging in N_2_, vacuum, and an air atmosphere, measured in a temperature range of 20 to 300 K.

For the pristine sample, no evident phase transition is observed in the PL spectra upon increasing the temperature (Figure 5a). The absence of phase transition at low temperature has also been observed in MAPbBr_3_ quantum dots and thin film [25,32]. As the measuring temperature increases, the PL emission of the pristine sample and samples after aging in different atmospheres all display blueshift, constituting the typical property of lead halide perovskite. This positive correlation between the band gap energy (E_g_) and temperature has been attributed to the antibonding nature of band-edge states [21] or the large temperature coefficient of lattice expansion [33]. Under the former assumption, the different behaviors between the valence band maxima (VBM) and conduction band minima (CBM) with changes in temperature cause the widening of E_g_. Upon an increase in temperature and lattice dilation, covalent Pb 6s and Br 5p antibonding interactions at VBM are weakened, lowering the VBM potential energy, while the CBM potential energy changes slightly. As a net effect, E_g_ increases [21]. Under the latter assumption, the large temperature coefficient of lattice expansion plays a much more significant role than the electron-phonon interactions does [33]. To obtain a unified understanding of the fundamental origins, further investigations are required.

**Figure 6:**
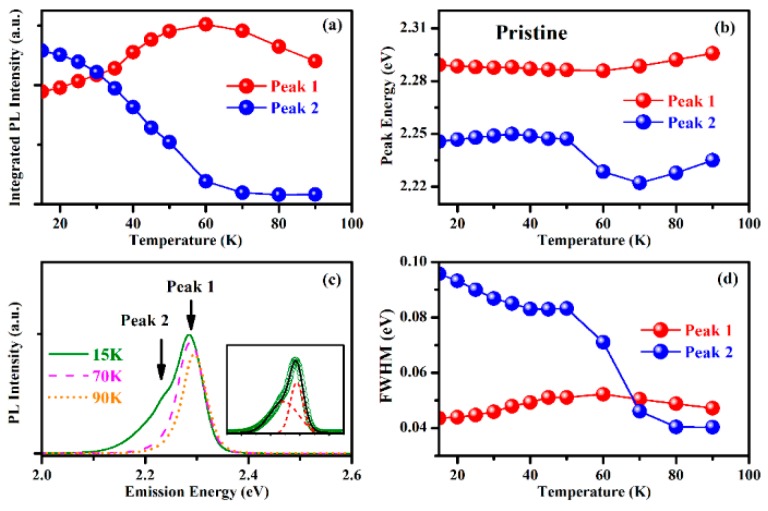
The comparison of two Gaussian fitted peaks from PL spectra of pristine MBNCs-PVDF: (**a**) Integrated PL intensity; (**b**) PL emission peak energy; and (**d**) FWHM of the fitted peaks obtained by two-peak Gaussian fitting. (**c**) PL spectra at 15 K, 70 K, and 90 K of pristine MBNCs-PVDF, illustrating the evolution of multipeak PL emission at low temperature, with the inset showing the two-peak Gaussian fitting result for the PL spectrum at 15 K.

Below 70 K, the PL spectra of pristine MBNCs-PVDF show a shoulder in the low-energy tail (Figure 5a and Figure 6c), which has also been demonstrated in thin films and single crystals of MAPbBr_3_ and attributed to trap emission from excitons bound by shallow defects before recombination [25]. Through Gaussian fitting, we extract two emission peaks, those for free exciton emission (peak 1) and trap emission (peak 2), from spectra at 15 K to 90 K (Figure 6c).

The integrated PL intensity of trap emission decreases as the temperature increases and becomes insignificant above 70 K (Figure 6a), illustrating the thermal release of bound excitons from defects [25,34]. Meanwhile, the integrated PL intensity of free exciton emission increase from 15 K and reaches a maximum at 70 K (Figure 6a). This increase originates from the thermal activation of trapped carriers, which overcomes the shallow energy barriers and contribute to radiative recombination [25,34]. The surface of MBNCs-PVDF directly contacts the PVDF matrix without the protection of capping surfactants, generating additional interfacial defect states that contribute to defect-related trap emission [25,32,34]. After the thermal release of carriers trapped in shallower states, peak 2 is dominated by the carriers from deeper states, causing the sudden red shift of peak energy of peak 2 between 50 K and 70 K (Figure 6b). Below 100 K, the peak energy of peak 1 of pristine sample varies within a small range (Figure 3b), which may originate from the thermal activation of trapped carriers. The enhancement of the integrated PL intensity at approximately 70 K is also observed in the samples aged in the three atmospheres (Figure 5b–d). The higher energy peak (peak 1), obtained through two-peak Gaussian fitting of PL spectra from 15 K to 90 K, exhibits maxima at 50 K, 60 K, and 70 K after aging in N_2_ (Figure S1a), vacuum (Figure S2a), and air (Figure S3a), respectively.

## 4. Discussion

Starting from the light-induced cleavage of the C–N bond in MA [14,17,35], we propose that the formation of NH_4_Pb_2_Br_5_ consists of two steps. First, MAPbBr_3_ decomposes to NH_4_Br, PbBr_2_, and hydrocarbon fragments (–CH_2_–), CH_3_NH_3_PbBr_3_ → NH_4_Br + PbBr_2_ + (–CH_2_–). Then, NH_4_Br reacts with PbBr_2_ to form a new NH_4_Pb_2_Br_5_ phase, NH_4_Br + 2PbBr_2_ → NH_4_Pb_2_Br_5_. A similar phase transformation in the inorganic perovskite CsPbBr_3_ has been reported, originating from ligand ratio tuning [36] or annealing above 423 K [37]. The existence of the intermediate product PbBr_2_ has been identified by XRD, as mentioned above (Figure 3a–c). NH_4_Br, another intermediate product, has also been identified from the peaks at 21.5°, 30.7°, and 37.8° in the XRD pattern, corresponding to reflections from the (001), (111), and (201) crystal planes, respectively. However, as illustrated in the zoomed-in XRD (Figure S4), these peaks overlap with those from the (110) and (200) crystal planes of MAPbBr_3_ and the (040) crystal plane of PbBr_2_. Though difficult to distinguish, the trace of NH_4_Br is revealed in the detailed XRD spectra at approximately 38° (Figure S5). The high-degree edge of the peak at 37.7°, corresponding to the (040) crystal plane of PbBr_2_, is broadened, which is possibly due to the contribution of the reflections from the (201) crystal plane of NH_4_Br.

One may also propose another possible product of this tetragonal phase, MAPb_2_Br_5_, resembling the reaction mechanism of CsPb_2_Br_5_ [37]. Starting from the decomposition of the initial material (MAPbBr_3_ → PbBr_2_ + MABr), PbBr_2_ reacts with MAPbBr_3_ to form MAPb_2_Br_5_ (PbBr_2_ + MAPbBr_3_ → MAPb_2_Br_5_) with a NH_4_Pb_2_Br_5_-like structure. However, the calculated XRD pattern of MAPb_2_Br_5_ does not match the diffraction peaks of MBNCs-PVDF after aging (Figure S6). Furthermore, the first decomposition reaction is thermodynamically disfavored [17], and XRD peaks from MABr (Figure 3f) cannot be found (Figure 3a–c). Therefore, we can exclude the formation of the MAPb_2_Br_5_ phase.

To separate the contribution between illumination and temperature, the MBNCs-PVDF samples are aged at a temperature of 300 K under the same 368 nm UV light illumination with an intensity of 5.6 mW/cm^2^ and aged at the temperature of 323 K without UV light illumination for the same time duration, 24 h, in three different atmospheres, respectively. After aging at 300K under UV light illumination (Figure S7) or at 323 K without UV light illumination (Figure S8), the XRD patterns are identical to that of the pristine sample and without the XRD peaks from NH_4_Pb_2_Br_5_. Therefore, the single stress of UV light or temperature cannot cause the generation of NH_4_Pb_2_Br_5_, whereas the synergistic effect from them cause the decomposition of MAPbBr_3_ and the generation of NH_4_Pb_2_Br_5_.

After aging at 300 K under UV light illumination (Figure S9), the PL intensities of MBNCs-PVDF aged in N_2_ and vacuum only change slightly, whereas the PL intensity of MBNCs-PVDF aged in air increase around four times. After aging at 323 K without UV light illumination (Figure S10), the PL intensities of MBNCs-PVDF aged in N_2_ and vacuum decrease to 40% of that of the pristine sample, and this value for the sample aged in air is 77%. Thus, the temperature predominately contributes to the significant fluorescence quenching in MBNCs-PVDF under the co-stresses of temperature and UV illumination that mimic operating conditions. Therefore, enhancing thermal stability acts as the most important task for MBNCs-PVDF. It is also worthwhile to mention that the change of PL intensities after aging in air is smaller than those aged in N_2_ and vacuum atmospheres. This may originate from the inactivation of traps in MAPbBr_3_ NCs by the limited amount of oxygen molecules [38,39], which permeate into PVDF and reach the surface of NCs, but further research is needed to obtain a detailed understanding.

From the XRD patterns, we observe that the NH_4_Pb_2_Br_5_ and its by-products exist in MBNCs-PVDF after aging, regardless of the type of the atmosphere they are in. That is, the MAPbBr_3_ NCs undergo the similar dissociation pathway in different atmospheres, under co-stresses of UV illumination and temperature. Therefore, we propose that the above-mentioned dissociation is a general mechanism of MAPbBr_3_ NCs. Furthermore, in order to improve the stability of perovskite, it is essential to investigate the reaction process of NH_4_Pb_2_Br_5_ in future works.

The change on optical absorption of the polymer matrix PVDF itself under the co-stresses of UV illumination and temperature could distort the PL of MBNCs-PVDF. The enhancement on the optical absorption of polymer matrix PVDF in the wavelength range of PL from NCs, i.e., 500 nm to 600 nm (Figure 2c), will reduce the measured PL intensity of MBNCs-PVDF even when the intrinsic emission from NCs remains the same, whereas the reduction on the optical absorption of the polymer matrix in that wavelength range will increase the PL intensity of MBNCs-PVDF. If the optical absorption of polymer matrix PVDF changes asymmetrically in that wavelength range, the measured PL spectra of MBNCs-PVDF would deviate from the intrinsic spectra of NCs, and the obtained peak energy and FWHM would be incorrect. Therefore, to exclude the influence from the degradation of polymer matrix PVDF itself, we compare the optical absorption of the pure polymer matrix PVDF film with the same thickness, prepared by using the same spin-coating method in Section 2, before and after 24 h aging in N_2_, vacuum and air, separately, for which the stresses are identical: 368 nm UV light illumination with an intensity of 5.6 mW/cm^2^ under a temperature of 323 K. Compared with that of the pristine pure PVDF film, there exits little changes in the absorption spectra of pure PVDF films after aging, under the same co-stresses of MBNCs-PVDF in different atmospheres (Figure S11). Therefore, in this work, the influence of degradation of PVDF itself on the PL of MBNCs-PVDF, and on the results of spectral analysis, could be excluded.

The densities of interface trap states of the aged samples are far higher than that of the pristine sample according to the fitting results from temperature-dependent PL spectra (Tables S2 and S3). Since the higher density of interface trap states in the aged samples, it is hard for the thermal activation to exhaust the carriers trapped in the interface defects, therefore, the sudden red shift of peak 2 between 50 K and 70 K is not observed in the aged samples (Figure S1b, Figure S2b, and Figure S3b). For an individual NC, its exterior are more susceptible to decomposition than interior due to the prior exposure to UV light; furthermore, according to the larger interface trap states of aged sample, we propose that dissociation mainly occurs at the surface area of NCs. Therefore, the co-stresses result in the generation of NH_4_Pb_2_Br_5_ and large amount of accompanying interface defects at the surface area of NCs. The existence of large amount of interface defects dissipates carriers and causes the reduction of PL intensity of the aged MBNCs-PVDFs.

## 5. Conclusions

We have demonstrated the generation of NH_4_Pb_2_Br_5_ from the MAPbBr_3_ NC embedded in PVDF matrix when it is subjected to UV illumination and a temperature of 323 K. This study is the first report of this new mechanism in MAPbX_3_. This decomposition could not be prevented by removing the water and oxygen from the atmosphere, as it could also occur in pure N_2_ and even in vacuum. Therefore, to evaluate the performance of perovskite NCs in optoelectronic applications, researchers should employ aging tests under combined external stresses, instead of just the individual one. It is necessary to develop new routes, besides encapsulation, to mitigate the intrinsic decomposition and the enhance stability of perovskite NCs.

## Supplementary Materials

The following are available online at https://www.mdpi.com/2079-4991/9/8/1158/s1, Figure S1: The comparison of two Gaussian fitted peaks from PL spectra of MBNCs-PVDF after aging in N_2_, Figure S2: The comparison of two Gaussian fitted peaks from PL spectra of MBNCs-PVDF after aging in vacuum, Figure S3: The comparison of two Gaussian fitted peaks from PL spectra of MBNCs-PVDF after aging in air, Figure S4, S5 and S6: X-ray diffraction (XRD) patterns of MAPbBr_3_ NCs embedded in PVDF film, Figure S7: X-ray diffraction patterns of MAPbBr_3_ NCs embedded in PVDF film after aging in three different atmospheres at a temperature of 300 K under 368 nm UV light illumination, Figure S8: X-ray diffraction patterns of MAPbBr_3_ NCs embedded in PVDF film after aging in three different atmospheres at a temperature of 300 K without UV light illumination, Figure S9: Normalized PL spectra of MBNCs-PVDF after aging in three different atmospheres at a temperature of 300 K under 368 nm UV light illumination, Figure S10: Normalized PL spectra of MBNCs-PVDF after aging in three different atmospheres at a temperature of 323 K without UV light illumination, Figure S11: Absorption spectra of pure PVDF film after aging in three different atmospheres at a temperature of 300 K without UV light illumination, comparing with that of the pristine sample, Figure S12: (a–d) The FWHM of PL spectra as a function of temperature, from 100 K to 300 K, of MBNCs-PVDF combined with the fitted curve using Equation S1. (e–h) The integrated PL intensity as a function of temperature combined with the fitted curve using Equation S2. The corresponding values of $E_{LO}$ fitted from (a–d) are used during the fitting in (e–h), Figure S13. The integrated PL intensity as a function of temperature combined with the fitted curve using Equation S3.
