# Supplementary material for: Stability of Hybrid Organic-Inorganic Perovskite CH3NH3PbBr3 Nanocrystals under Co-Stresses of UV Light Illumination and Temperature
Source: Nanomaterials (Basel). 2019 Aug 13;9(8):1158. doi: 10.3390/nano9081158 (PMC6724138; doi:10.3390/nano9081158)
Supplement: Supplementary file 1 [file nanomaterials-09-01158-s001.pdf]

Supporting information

Stability of hybrid organic-inorganic perovskite $CH_3NH_3PbBr_3$ nanocrystals under co-stresses of UV light illumination and temperature

Weijie Guo [1], Nan Chen [1], Binbin Xu [2], Yijun Lu [1], Bin Li [3], Tingzhu Wu [1], Qijin Cheng [1], Yang Li [4], Jin Chen [4], Yue Lin [1,*] and Zhong Chen [1,5,*]

1 Fujian Engineering Research Center for Solid-State Lighting, Collaborative Innovation Center for Optoelectronic Semiconductors and Efficient Devices, Department of Electronic Science, Xiamen University, Xiamen 361005, China; wjguo@stu.xmu.edu.cn (W.G.); nanchen@stu.xmu.edu.cn (N.C.); yjlu@xmu.edu.cn (Y.L.); wutingzhu@xmu.edu.cn (T.W.)

2 College of Chemistry and Chemical Engineering, Xiamen University, Xiamen 361005, China; xubinbin@xmu.edu.cn (B.X.)

3 School of Science, Nanjing University of Posts and Telecommunications, Nanjing, 210023, China; libin@njupt.edu.cn (B.L.)

4 College of Energy, Xiamen University, Xiamen 361102, China; qijin.cheng@xmu.edu.cn (Q.C.); liyxmu@126.com (Y.L.); jinchen@xmu.edu.cn (J.C.)

5 State Key Laboratory for the Physical Chemistry of Solid Surfaces, Xiamen University, Xiamen 361005, China

* Correspondence: yue.lin@xmu.edu.cn (Y.L.); chenz@xmu.edu.cn (Z.C.); Tel.: +86-592-2181712 (Y.L. & Z.C.)

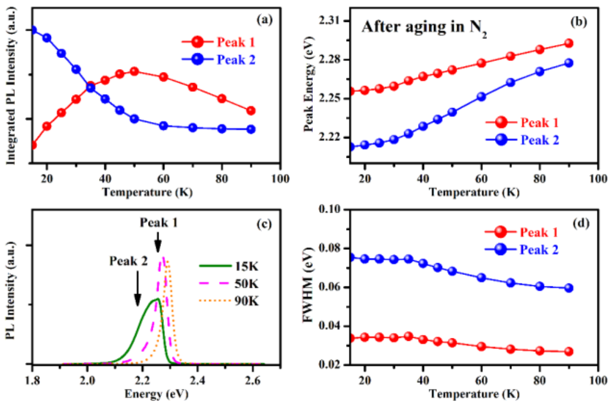

Figure S1. The comparison of two Gaussian fitted peaks from PL spectra of MBNCs-PVDF after aging in N_2: (**a**) integrated PL intensity; (**b**) PL emission peak energy and (**d**) FWHM of the fitted peaks obtained by two-peak Gaussian fitting. (**c**) PL spectra at 15 K, 50 K and 90 K of MBNCs-PVDF after aging in N_2, illustrating the evolution of multipeak PL emission at low temperature.

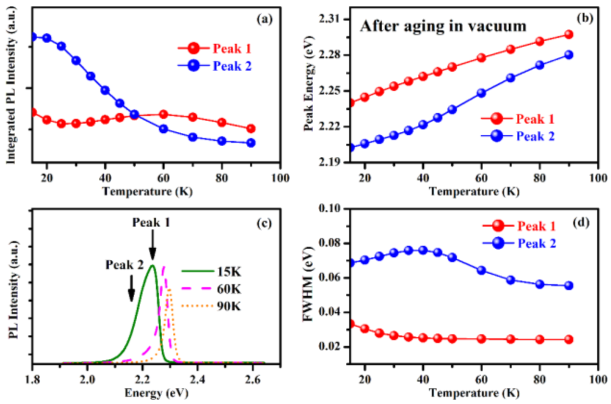

Figure S2. The comparison of two Gaussian fitted peaks from PL spectra of MBNCs-PVDF after aging in vacuum: (**a**) integrated PL intensity; (**b**) PL emission peak energy and (**d**) FWHM of the fitted peaks obtained by two-peak Gaussian fitting. (**c**) PL spectra at 15 K, 60 K and 90 K of MBNCs-PVDF after aging in vacuum, illustrating the evolution of multipeak PL emission at low temperature.

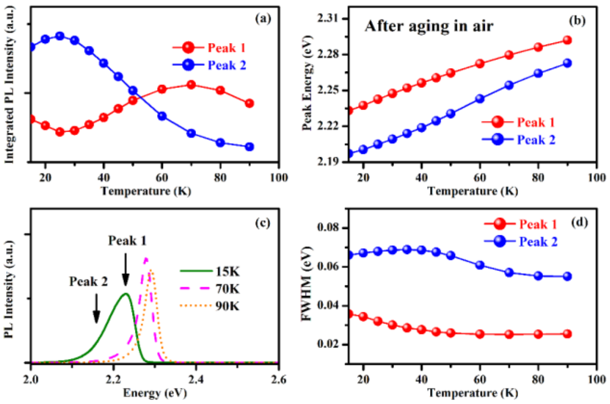

Figure S3. The comparison of two Gaussian fitted peaks from PL spectra of MBNCs-PVDF after aging in air: (**a**) integrated PL intensity; (**b**) PL emission peak energy and (**d**) FWHM of the fitted peaks obtained by two-peak Gaussian fitting. (**c**) PL spectra at 15 K, 70 K and 90 K of $MAPbBr_3$ NCs embedded in PVDF film after aging in air, illustrating the evolution of multipeak PL emission at low temperature.

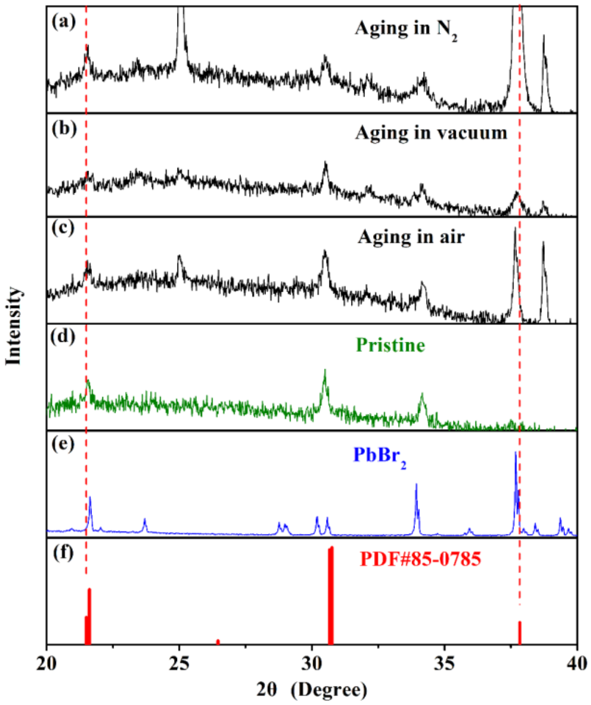

Figure S4. X-ray diffraction (XRD) patterns of $MAPbBr_3$ NCs embedded in PVDF film: (**a**) after aging in N_2; (**b**) after aging in vacuum; (**c**) after aging in an air atmosphere and (**d**) pristine. For reference, (**e**) provides XRD of $PbBr_2$ and (**f**) provides a standard XRD pattern of NH_4Br (PDF#85-0785, Tetragonal, P4/nmm). Vertical lines indicate diffraction peaks at 21.5° and 37.8° correspond to reflections from the (001) and (201) crystal planes of NH_4Br.

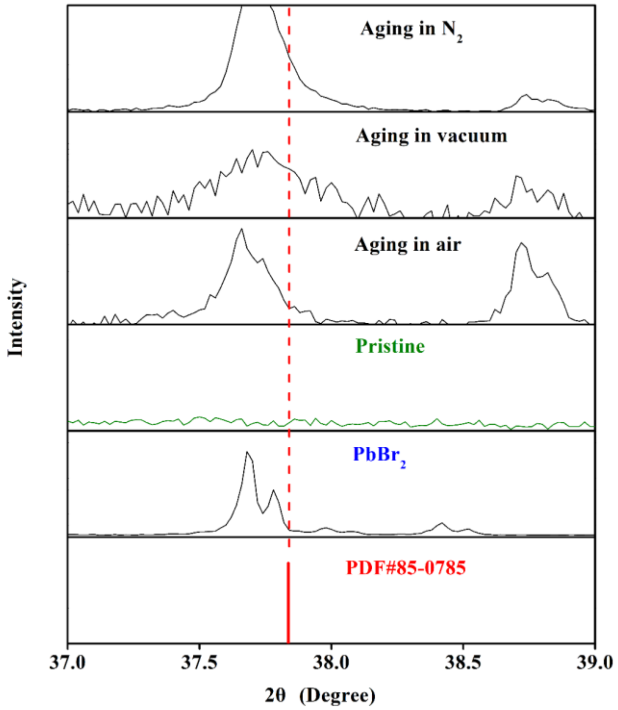

Figure S5. X-ray diffraction (XRD) patterns of $MAPbBr_3$ NCs embedded in PVDF film: (**a**) after aging in N_2; (**b**) after aging in vacuum; (**c**) after aging in air atmosphere and (**d**) pristine. For reference, (**e**) provides XRD of $PbBr_2$ and (**f**) provides a standard XRD pattern of NH_4Br (PDF#85-0785, Tetragonal, P4/nmm). Vertical dashed lines indicate the diffraction peak at 37.8° corresponding to the reflection from (201) crystal planes of NH_4Br.

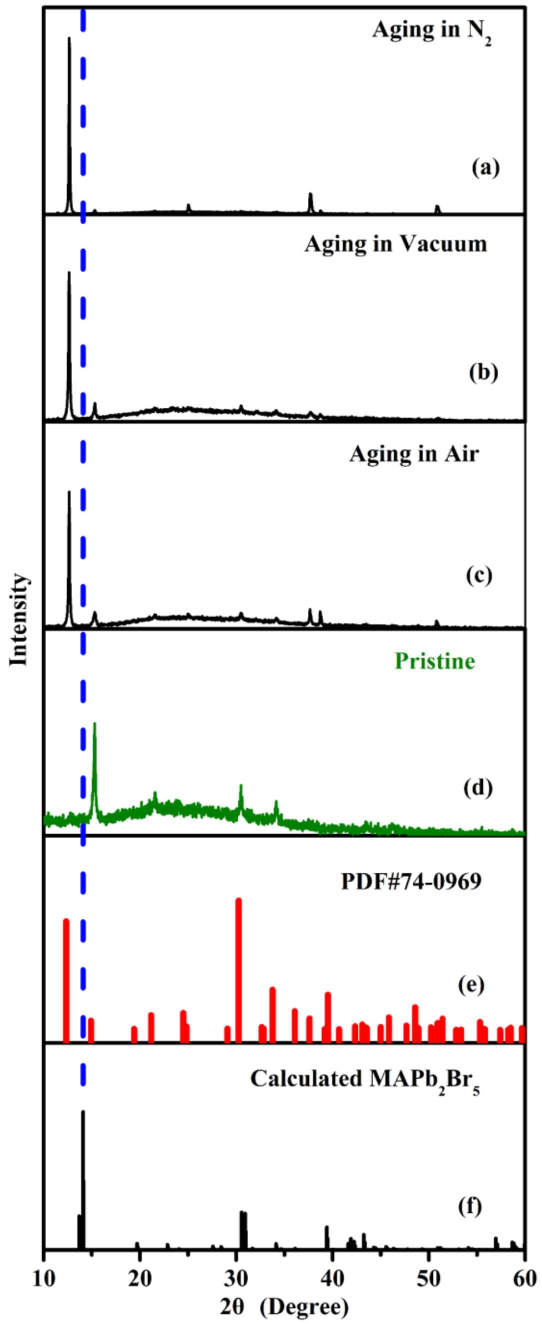

Figure S6. X-ray diffraction patterns of $MAPbBr_3$ NCs embedded in PVDF film: (**a**) after aging in N_2; (**b**) after aging in vacuum; (**c**) after aging in air atmosphere and (**d**) pristine sample. For reference, (**e**) and (**f**) provide standard XRD pattern of $NH_4Pb_2Br_5$ (PDF#74-0969) and calculated pattern of $CH_3NH_3Pb_2Br_5$ ($MAPb_2Br_5$), respectively.

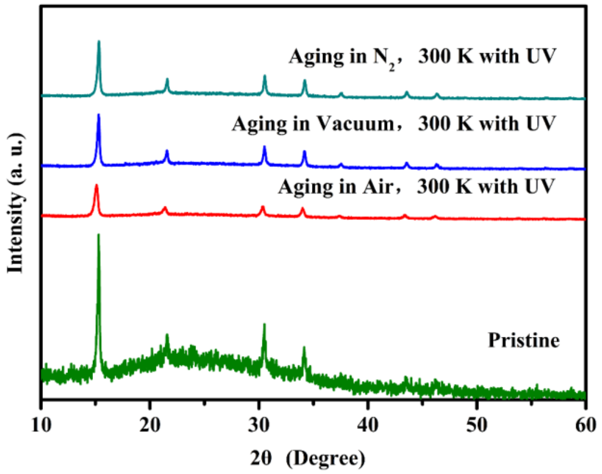

Figure S7. X-ray diffraction patterns of $MAPbBr_3$ NCs embedded in PVDF film after aging in three different atmospheres at a temperature of 300 K under 368 nm UV light illumination with an intensity of 5.6 mW/cm^2, comparing with that of the pristine sample.

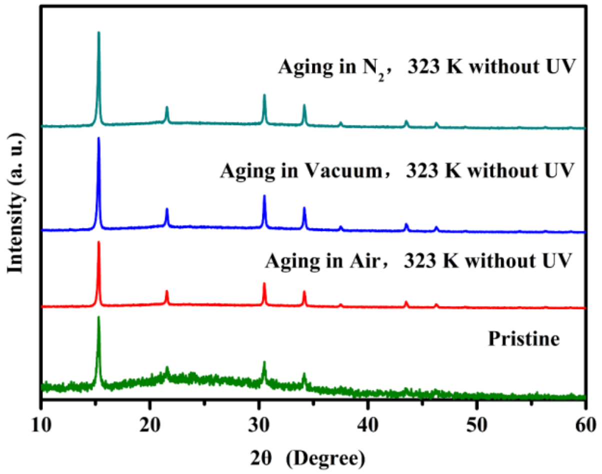

Figure S8. X-ray diffraction patterns of $MAPbBr_3$ NCs embedded in PVDF film after aging in three different atmospheres at a temperature of 300 K without UV light illumination, comparing with that of the pristine sample.

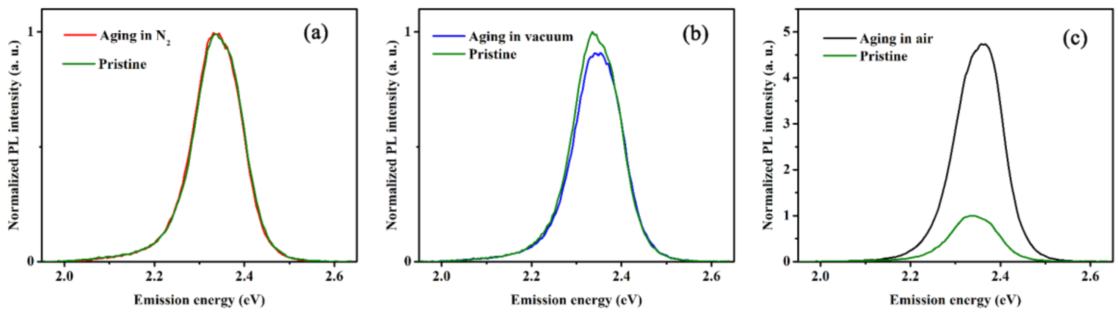

Figure S9. Normalized PL spectra of MBNCs-PVDF after aging in three different atmospheres at a temperature of 300 K under 368 nm UV light illumination with an intensity of 5.6 mW/cm^2. Normalized to the PL peak of the pristine MBNCs-PVDF. (**a**) PL spectra of the pristine MBNCs-PVDF and the sample after aging in N_2; (**b**) PL spectra of the pristine MBNCs-PVDF and the sample after aging in vacuum and (**c**) PL spectra of the pristine MBNCs-PVDF and the sample after aging in air atmosphere.

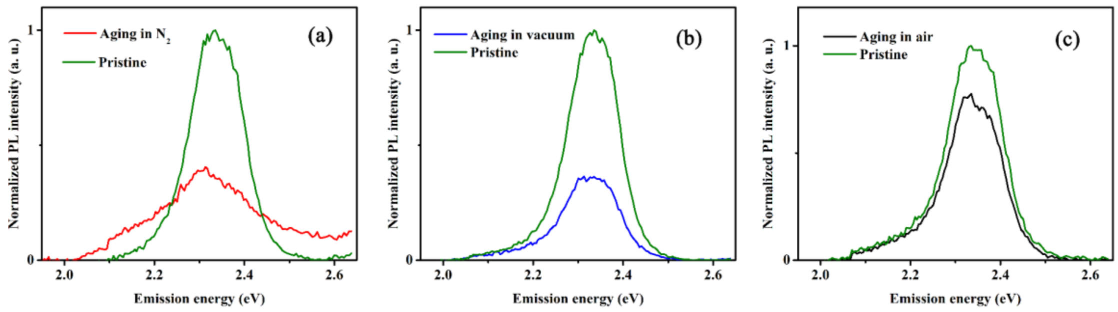

Figure S10. Normalized PL spectra of MBNCs-PVDF after aging in three different atmospheres at a temperature of 323 K without UV light illumination. Normalized to the PL peak of the pristine MBNCs-PVDF. (**a**) aging in N_2; (**b**) aging in vacuum and (**c**) aging in air atmosphere.

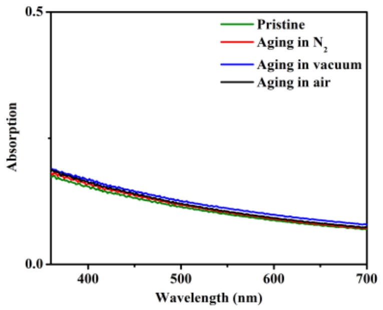

Figure S11. Absorption spectra of pure PVDF film after aging in three different atmospheres at a temperature of 300 K without UV light illumination, comparing with that of the pristine sample. The temperature and 368 nm UV light illumination density during aging are 323 K and 5.6 mW/cm^2.

We further analyzed the electron-phonon coupling in MBNCs-PVDF, from the temperature-dependent PL broadening. The relationship between the FWHM of the PL spectra and temperature was determined to be [1]:

$$\Gamma(\mathrm{T}) = \Gamma_0 + \gamma_{ac}T + \gamma_{LO}\frac{1}{e^{E_{LO}/k_BT} - 1} \tag{S1}$$

where Γ_0 denotes the temperature-independent inhomogeneous broadening of the FWHM, γ_{ac} the coupling coefficient between exciton and acoustic phonon, γ_{LO} the coupling coefficient between exciton and longitudinal optical phonon (LO-phonon), E_{LO} the LO-phonon energy, k_B and Boltzmann's constant.

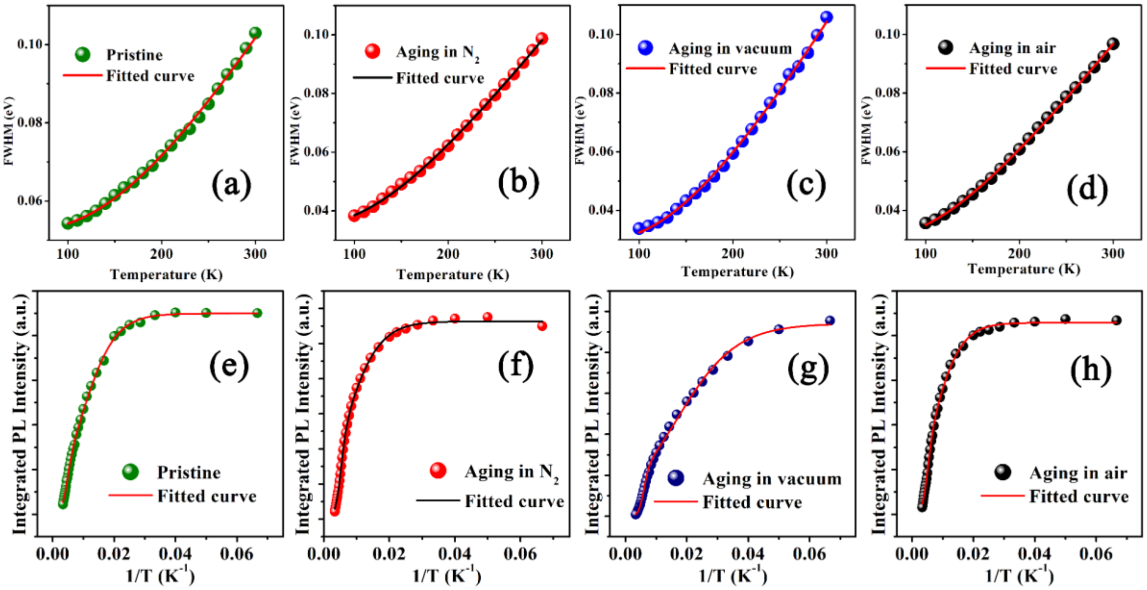

Figure S12. (**a-d**) The FWHM of PL spectra as a function of temperature, from 100 K to 300 K, of MBNCs-PVDF combined with the fitted curve using Equation S1. (**e-h**) The integrated PL intensity as a function of temperature combined with the fitted curve using Equation S2. The corresponding values of E_{LO} fitted from (**a-d**) are used during the fitting in (**e-h**).

By extracting and fitting FWHM of PL spectra above 100 K, we exclude the additional broadening of PL spectra due to emission from two types of excitons below 90 K [2]. FWHM versus temperature and curves fitted by equation 1 are plotted in Figures S12a-S12d. The fitted results of are collected in Table S1. The fitted E_{LO} of pristine MBNCs-PVDF equals 46 meV, which is consistent with the previously reported value of 42.2 meV for 3.3 nm $MAPbBr_3$ NCs [3], and larger than the value of 24.9 meV for 10 nm NCs [1].

Furthermore, taking into account the contribution of thermally activated trapped carriers to the enhancement of PL, thermal dissociation of exciton, and thermal escape of carriers, the temperature dependence of integrated PL intensity is given by [1,4]:

$$I_{PL}(T) = \frac{N_0 + N_0' e^{-\Delta E/k_BT}}{1 + Be^{-E_b/k_BT} + C(e^{E_{LO}/k_BT} - 1)^{-m}} \tag{S2}$$

where N_0' denotes the density of interface trap states, ΔE activation energy of interface trap states, $I_{PL}(T)$ the integrated PL intensity at temperature T, N_0 the initial carrier population, E_b the activation energy for exciton dissociation, m the number of longitudinal optical phonon (LO-phonon) for assisted thermal escape of carriers, E_{LO} LO-phonon energy, k_B Boltzmann's constant, B and C fitting parameters.

Table S1. Extracted photoluminescence linewidth broadening parameters.

**Sample**	Γ_0 **(meV)**	γ_{ac} **(meV)**	γ_{LO} **(meV)**	E_{LO} **(meV)**
Pristine	50.8±1.8	0.0245±0.015	214.8±14.7	46.0±1.9
Aging in N_2	34.7±1.4	0.0078±0.013	177.7±8.1	35.2±1.0
Aging in vacuum	28.9±0.3	0.0097±0.012	273.1±2.1	40.4±1.9
Aging in air	30.5±1.6	0.0056±0.016	158.8±8.3	32.1±0.9

Table S2. The parameters fitted by Equation S2.

**Sample**	E_b **(meV)**	m	ΔE **(meV)**	N_0'
Pristine	103.1±3.1	0.35±0.01	18.7±2.2	36±13
Aging in N_2	109.3±4.9	0.47±0.19	17.4±3.8	916±41
Aging in vacuum	108.7±4.1	0.23±0.01	17.0±1.6	370±26
Aging in air	108.5±3.6	0.56±0.02	19.1±2.7	280±32

Using the extracted E_{LO} by Equation 1 in Figures S12a- S12d, we fit the temperature-dependent integrated PL intensity by Equation S2 (Figures S12e- S12h). Assuming that there exist trap states above the exciton ground state, we replace the LO-phonon-assisted thermal escape term in Equation S2 with another exponential term which denotes the thermally-activated trap of excitons [5], and then rewrite the temperature dependence of integrated PL intensity as:

$$I_{PL}(T) = \frac{N_0 + N_0' e^{-\Delta E/k_B T}}{1 + B e^{-E_b/k_B T} + D e^{-E_a/k_B T}} \quad (3)$$

In Equation S3, D is a fitting parameter, E_a the activation energy of thermally activated trap. The fitted curves by Equation S3 are exhibited in Figures S13a- S13d, and the fitted results are collected in Table S3. For the same sample, the values of E_b, or ΔE, fitted by Equation S2 (Table S2) and S3 (Table S3) are consistent. The values of E_a (Table S3) fitted by Equation S3 are close to the products of m (Tables S2) and E_{LO} (Table S1) of each sample, respectively.

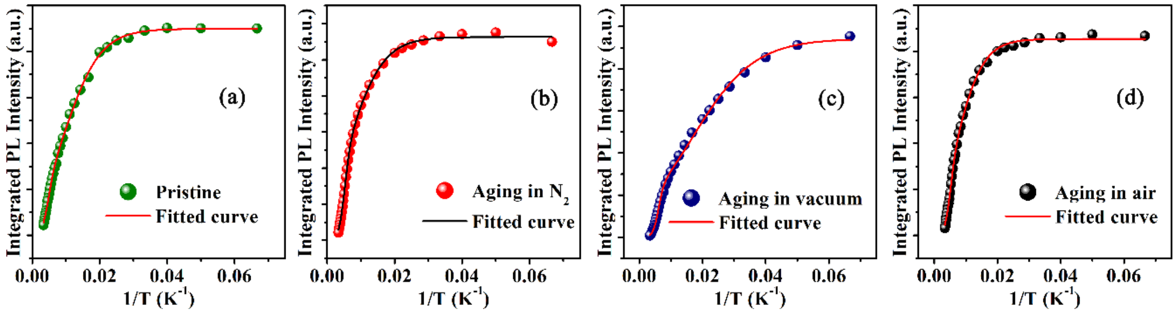

Figure S13. The integrated PL intensity as a function of temperature combined with the fitted curve using Equation S3.

Table S3. Exciton binding energy and other parameters fitted by Equation S3.

Sample	E_b (meV)	E_a (meV)	ΔE (meV)	N'_0
Pristine	102.1±2.7	16.2±2.2	17.1±3.7	62±12
Aging in N_2	108.9±3.8	16.9±8.7	16.9±3.8	911±97
Aging in vacuum	108.3±4.5	8.9±0.3	18.6±2.2	374±33
Aging in air	109.3±3.1	20.1±5.6	21.7±3.6	329±30

The exciton binding energy (E_b) of MBNCs-PVDF after aging become larger than that of pristine MBNCs-PVDF (Table S3), suggesting the enhancement of quantum confinement after aging [1,3,6].

References

1. Woo, H.C.; Choi, J.W.; Shin, J.; Chin, S.H.; Ann, M.H.; Lee, C.L. Temperature-Dependent Photoluminescence of $CH_3NH_3PbBr_3$ Perovskite Quantum Dots and Bulk Counterparts. *J. Phys. Chem. Lett.* **2018**, *9*, 4066-4074, doi:10.1021/acs.jpclett.8b01593.
2. Wright, A.D.; Verdi, C.; Milot, R.L.; Eperon, G.E.; Pérez-Osorio, M.A.; Snaith, H.J.; Giustino, F.; Johnston, M.B.; Herz, L.M. Electron–Phonon Coupling in Hybrid Lead Halide Perovskites. *Nat. Commun.* **2016**, *7*, 11755, doi:10.1038/ncomms11755.
3. Zhang, F.; Zhong, H.; Chen, C.; Wu, X.; Hu, X.; Huang, H.; Han, J.; Zou, B.; Dong, Y. Brightly Luminescent and Color-Tunable Colloidal $CH_3NH_3PbX_3$ (X = Br, I, Cl) Quantum Dots: Potential Alternatives for Display Technology. *ACS Nano* **2015**, *9*, 4533-4542, doi:10.1021/acsnano.5b01154.
4. Jing, P.; Zheng, J.; Ikezawa, M.; Liu, X.; Lv, S.; Kong, X.; Zhao, J.; Masumoto, Y. Temperature-Dependent Photoluminescence of CdSe-Core CdS/CdZnS/ZnS-Multishell Quantum Dots. *J. Phys. Chem. C* **2009**, *113*, 13545-13550, doi:10.1021/jp902080p.
5. De Giorgi, M.L.; Perulli, A.; Yantara, N.; Boix, P.P.; Anni, M. Amplified Spontaneous Emission Properties of Solution Processed $CsPbBr_3$ Perovskite Thin Films. *J. Phys. Chem. C* **2017**, *121*, 14772-14778, doi:10.1021/acs.jpcc.7b00854.
6. Zheng, K.; Zhu, Q.; Abdellah, M.; Messing, M.E.; Zhang, W.; Generalov, A.; Niu, Y.; Ribaud, L.; Canton, S.E.; Pullerits, T. Exciton Binding Energy and the Nature of Emissive States in Organometal Halide Perovskites. *J. Phys. Chem. Lett.* **2015**, *6*, 2969-2975, doi:10.1021/acs.jpclett.5b01252.
